# POWGEN: rebuild of a third-generation powder diffractometer at the Spallation Neutron Source

**DOI:** 10.1107/S160057671901121X

**Published:** 2019-10-01

**Authors:** Ashfia Huq, Melanie Kirkham, Peter F. Peterson, Jason P. Hodges, Pamela S. Whitfield, Katharine Page, Thomas Hűgle, Erik B. Iverson, Andre Parizzi, George Rennich

**Affiliations:** a Neutron Scattering Division, Oak Ridge National Laboratory, Oak Ridge, TN 37831-6475, USA; b Neutron Technology Division, Oak Ridge National Laboratory, Oak Ridge, TN 37831-6475, USA; c Excelsus Structural Solutions, Park Innovaare, 5234 Villigen, Switzerland

**Keywords:** neutron powder diffraction, POWGEN, *in situ*/*operando* measurements

## Abstract

The neutron powder diffractometer POWGEN at the Spallation Neutron Source has recently (2017–2018) undergone an upgrade which resulted in an increased detector complement along with a full overhaul of the structural design of the instrument. The current instrument has a solid angular coverage of 1.2 steradians and maintains the original third-generation concept, providing a single-histogram data set over a wide *d*-spacing range and high resolution to access large unit cells, detailed structural refinements and *in situ*/*operando* measurements.

## Abstract 2

This work describes the design principles and upgrade of the neutron powder diffractometer POWGEN at the Spallation Neutron Source.

## 1. Introduction   

Traditional time-of-flight (TOF) diffractometers use detector ‘banks’ at a few selected angles along with a broad wavelength range to produce a limited number of data sets of intensity versus wavelength at these few scattering angles. The angular range of these ‘banks’ is selected so that the peak widths from the combined scattering angles are the same, resulting in constant resolution data sets, albeit with limited *d*-spacing or elastic momentum transfer *Q* (= 2π/*d*) range. In contrast, a constant wavelength (CW) diffractometer at a reactor source can access longer *d* spacings and relatively low *Q*_max_ with a variable resolution (δ*d*/*d*). The third-generation concept of a TOF diffractometer was proposed at the 14th Meeting of the International Collaboration on Advanced Neutron Sources by Radaelli (1998 ▸). This concept maximizes the short bandwidth resulting from the 60 Hz operation at the Spallation Neutron Source (SNS) and an uninterrupted angular coverage afforded by a continuous detector locus to collect a single data set of intensity versus angle and time of flight. By design, POWGEN is a diffractometer that bridges the gap between traditional TOF and CW diffractometers, offering access to high *Q*, variable resolution as a function of *d* spacing and access to large *d* spacing by changing the wavelength band of operation.

The various components of this instrument starting from the moderator, choppers, guide and detectors were previously described by Huq *et al.* (2011 ▸). In this paper, we will describe the design philosophy in detail and its impact on data collection strategy for different types of scientific experiments conducted at the instrument. Scientific examples are provided to elucidate the versatile science for which this instrument can be used. We will also describe the gains offered by the rebuild and propose some future upgrades that might be considered.

## 2. Instrument upgrade descriptions and future upgrades   

**Figure 1:**
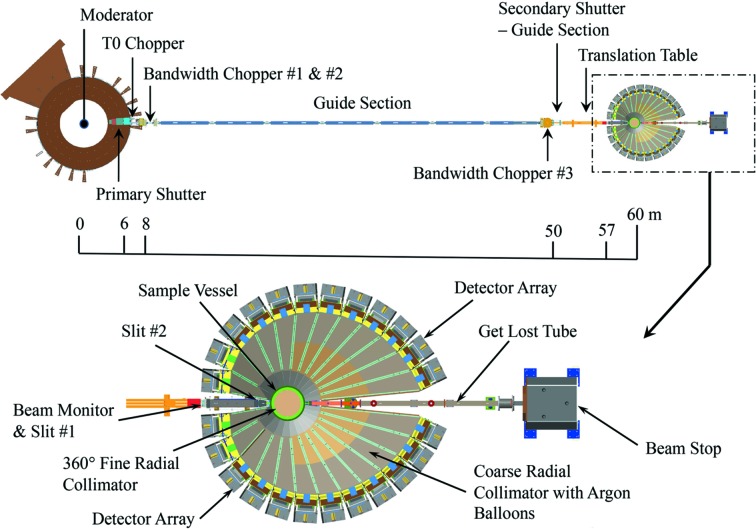
Schematic layout of the POWGEN instrument. The upgraded section of the instrument is magnified, showing details of each component.

POWGEN entered the user program at SNS in late 2010 with a somewhat limited detector coverage of 30–130° in 2Θ and most of the detectors in plane (12 modules of scintillator-based wavelength shifting fiber neutron detectors). The detector complement was gradually increased over the next couple of years to 30 modules. Significant improvement in instrument performance was also achieved through advances in event positioning algorithms and scintillators (Wang, 2016 ▸, 2015 ▸) to improve detector efficiency. Even with the various improvements since entering the user program, POWGEN was operating at a much lower performance level than envisioned in the original design. The instrument was unable to reach its full potential owing to the slow overall count rate and compromised engineering design that included shadowing of detectors, flexing of Al windows during evacuation of the tank *etc*. The prominent deficiencies addressed by this upgrade project reside in the instrument cave optics, which include all components downstream from the interchangeable guide located at 57 m from the core vessel insert as shown in Fig. 1 ▸. The neutron beam delivered from the 5 m interchangeable guides is within the instrument performance specification and thus the front end of the instrument, which includes the moderator, disc choppers and neutron guide, was not a part of this upgrade and remains unchanged.

Before the start of this project, most of the instrument components were built on only one half of the instrument, which included the detectors and the coarse and fine radial collimators. The sample vacuum vessel was built of aluminium with a tear-drop shape, with two 5 mm-thick Al windows. The previous design incorporated a steel shielding shroud that surrounded the vessel to support the coarse radial collimator assembly. This shroud structure blocked access to the downstream optics and masked the window. The new sample vacuum vessel is cylindrical and fabricated from ASTM A316L stainless steel, with 3 mm-thick aluminium windows, and incorporates two new high-efficiency turbo pumps. The vessel was also designed to support large sample environment ancillary equipment, such as a 5 T magnet. It is lined with vacuum-compatible 7 mm-thick reaction-bonded boron carbide on the inside surfaces and 5 mm Mirrobor (high boron content flexible neutron shielding purchased from Mirrotron) outside around the windows to reduce background.

The coarse radial collimator assembly is made of vertical and horizontal panels of B_4_C/fluoro­polymer-painted Al suspended from adjustable ceiling supports. The panels are constructed to form collimation cells for each detector column. To reduce the background contribution, the air in each cell is displaced by an Ar (ultra-high purity, 99.99%) filled ethyl­ene vinyl alcohol copolymer film balloon. The design allows easy access to the detectors for maintenance and reduces the shadowing on the detectors significantly. The array of detector stands was designed to allow a 2Θ coverage of 8–170° around the sample. The incident optics between the translation guides and the vessel were also redesigned to add two vacuum slits (one immediately following the translation guides and one just before the vessel) for improved beam spatial and divergence definition. In the standard configurations (as shown in Table 1 ▸) a beam size of 1 cm wide × 4 cm tall is used, but this can be adjusted as the experiment dictates. The maximum beam size that is achievable is 2 × 5.5 cm at a wavelength of 1.6 Å. However, the most intense part of the beam is contained within 1 × 4 cm. The new incident beam optics design incorporated bellows to extend the evacuated flight path and eliminate two upstream aluminium windows. An evacuated containment was also included in the design to accommodate, as a future option, a translator system that can move one or two 1.5 m focusing guides in and out of the beam position.

Finally, a 360° fine radial collimator was designed to provide full detector coverage with optimized structural column supports. The column supports were positioned to eliminate shadowing effects and align with the two fixed sample vessel support ribs. The fine radial collimator oscillates by ±2.00°. The blade foils are 12.5 µm-thick Kapton coated with boron carbide powder enriched to 96% ^10^B. The powder was mixed with a vacuum-compatible binder to achieve an areal density of B_4_C of 5.3 mg cm^−2^. The total coated blade thickness was 100 ± 10 µm. More details of the design can be found in the report by Huq & Rennich (2016 ▸). We have observed that this collimator is most effective in the 2Θ range 30–150° where the background is reduced by an order of magnitude. Slightly elevated background is observed in the data near the forward and backscattering angles.

**Figure 2:**
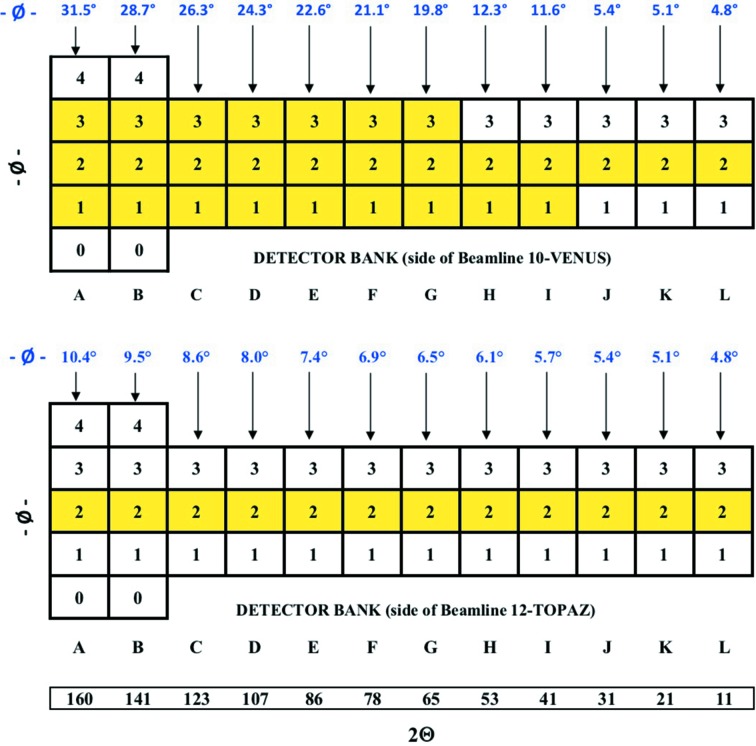
2Θ and ϕ (azimuthal angle at the centre of the outer detector of the column) locations of detectors have been folded out in two dimensions to show where the detector modules are installed. The 2Θ values shown are the centers of each detector stand.

The detector coverage was increased from 30 modules to 40 modules. The in-plane positions on both sides were filled (24 detectors) and the remaining 16 detectors were placed out of plane on the previously empty side of the instrument, as seen in Fig. 2 ▸. This choice was made as the divergence from the incident optics allows the highest resolution in the in-plane locations. The far out-of-plane locations in backscattering and forward scattering positions require higher spatial resolution in the vertical direction to follow the higher curvature of the Debye–Sherrer diffraction lines, thereby minimizing associated diffraction peak broadening. The detector array consists of wavelength shifting fiber (WLSF) scintillator detector modules, each with an active area of 38.5 × 77 cm and coarse pixel size of 5 × 55 mm (horizontal × vertical). At POWGEN, there are 20 version 2 and 20 version 3 detector modules, which differ in the mapping of vertical position-encoding fibers. Both are based around the same 32 single-anode photomultiplier tube channels for time-active discrimination and FPGA electronics board for signal processing. Version 3 fiber mapping is optimized for subpixel interpolation of the neutron event position in both the horizontal and vertical directions, whereas version 2 mapping is optimized for fine horizontal positioning only. Both versions are expected to ultimately have horizontal spatial resolutions of ∼3 mm and vertical spatial resolutions of 55 and ∼20 mm, respectively. Both version 2 and version 3 detectors are used in the current instrument.

Interpolation positioning is a future option for POWGEN. This necessitates a large increase in pixilation of the instrument and subsequent incorporation into all the instrument calibration, normalization and data reduction routines, and thus it requires further development work and testing. In addition, next-generation WLSF scintillator detector modules are currently under development which incorporate three 64-sensor multi-anode photomultiplier tubes yielding 192 electronic channels for a higher spatial resolution of 3 × 10 mm. It is envisioned that once 40 new-generation detector modules are constructed, all 80 detector positions can be filled in an optimal arrangement.

The upgrade resulted in a net gain of counts by 40% between 2016 and 2017. The *d*-spacing range of data that can be accessed in a single measurement has also increased substantially. For example, in 2016 one could collect a *d*-spacing range from 0.3 to 5.2 Å using a center wavelength of 1.066 Å at 60 Hz; after the upgrade a center wavelength of 0.8 Å allows diffraction data collection in the range 0.135–8.2 Å. A longer counting time (compared with that needed for Rietveld refinement) to acquire requisite statistics in this *d*-spacing range (corresponding to a momentum transfer, *Q*, range 0.7–47 Å^−1^) can also be used to carry out pair distribution function (PDF) analysis. Prior to the upgrade it was necessary to perform two independent measurements with two different wavelength bands to achieve the required low and high *Q* to generate total scattering data.

A future upgrade is expected to incorporate the higher-resolution detectors currently under development. There is also provision to add another 2 m guide section before the sample position, which would improve the high-intensity option for this instrument.

## 3. Data analysis considerations   

### 3.1. Mode of operation   

**Figure 3:**
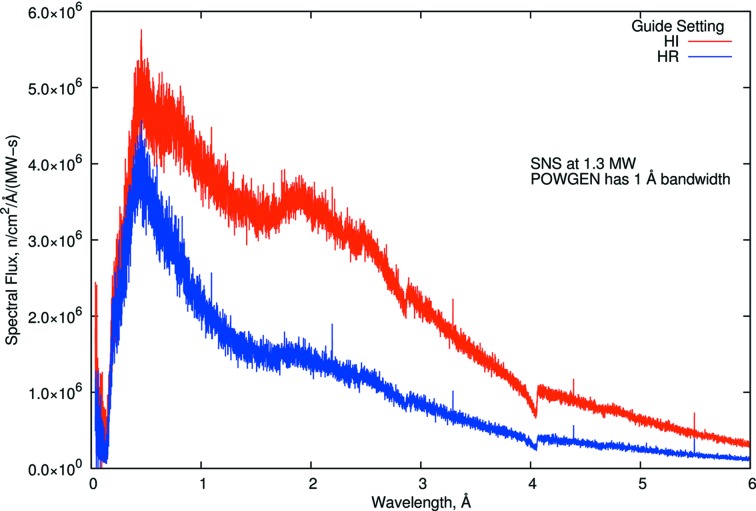
Spectral neutron flux at the POWGEN sample position for the high-intensity and high-resolution guide settings. Note that instrument chopper settings permit use of any 1 Å wavelength band.

At POWGEN, when the choppers are rotating at 60 Hz, the wavelength (λ) band available is ∼1 Å. The incident flux on the sample as a function of λ is shown in Fig. 3 ▸ across the extended λ range (0–6 Å). The instrument can be operated to provide the full bandwidth at reduced effective power, any 1 Å bandwidth at full power or an intermediate combination. The interchangeable guides offer the option of collecting data in high-resolution or high-intensity mode. Despite the presence of the *T*_0_ chopper, we observe a very small signal from the prompt pulse, as can be seen at long wavelengths at 1 Å intervals in Fig. 3 ▸. Approximately 50 µs of data are removed from the beginning of each 60 Hz frame before the data are time focused. As a result, although this instrument can operate at any λ region using the disc choppers, because of the need to calibrate the instrument in each configuration we have chosen several specific λ bands for standard operation. These pre-calibrated settings allow smooth operation of the beamline and offer the data range needed for most crystallographic problems. This also enables users to use all their beamtime to only collect data on their samples. Table 1 ▸ shows these standard settings of wavelengths and the *d* and *Q* ranges obtained from them. It should be noted that the modes lower than 60 Hz are rarely used owing to the associated loss of intensity and the fact that the 60 Hz operation is sufficient for most crystallographic experiments. Also, very few standard samples have reflections for the frame with λ centered at 4.797 Å to allow profile parameters to be easily verified.

**Figure 4:**
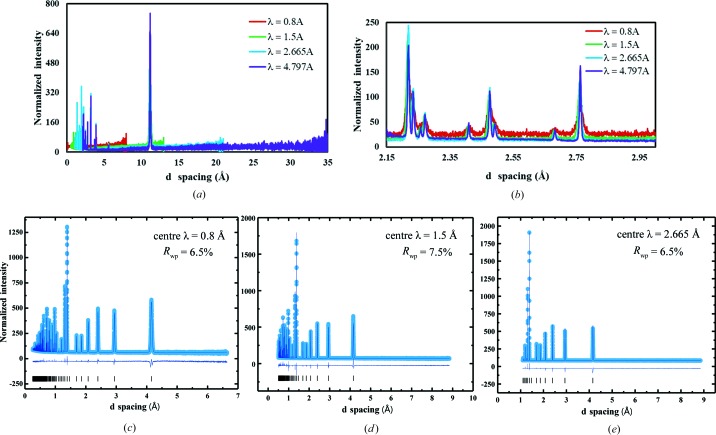
(*a*) Data collected at various different center wavelengths for CsLaNb_2_O_7_, showing the difference in data range, resolution and intensity as described in Table 1 ▸ for 60 Hz data only. Shorter wavelengths provide information at much shorter *d* spacing (higher *Q*). (*b*) Selected region of the data showing that the longer wavelengths provide better resolution and statistics. (*c*)–(*e*) Rietveld refinement of LaB_6_ (SRM 660b) using center wavelengths of 0.8, 1.5 and 2.665 Å in the high-resolution mode. The light-blue dots are the observed intensity, the navy-blue line is the calculated intensity, and also shown are the difference plot and tick markers. The values of full width at half-maximum reported in Fig. 6 ▸ are obtained from these fits. The overall goodness parameters for all three histograms are *R*_wp_ = 6.84% and χ^2^ = 4.48.

Although extreme *Q* is theoretically achievable, the count rates at very high and very low *Q* are negligible because of the guide design (see Fig. 3 ▸). Also, these ranges are valid for the sample changer where there is no physical obstruction from the device and may not be achievable for all sample environments. In Figs. 4 ▸(*a*) and 4(*b*), data sets for the four standard wavelength settings from a sample that has a long *d*-spacing peak (CsLaNb_2_O_7_) are shown. A zoomed-in region of the data clearly shows how resolution and intensities can vary from data collected for the same amount of time.

### 3.2. Data collection   

Data collection at POWGEN is implemented in the Linux-based *Experimental Physics and Industrial Control System* (*EPICS*) software (Dalesio *et al.*, 1991 ▸) with a graphical user interface through *Control System Studio* (Kasemir, 2007 ▸), a setup common across all beamlines at the Spallation Neutron Source. Data are collected in event mode (Peterson *et al.*, 2015 ▸), where each discrete neutron event is recorded individually and can be correlated with extensive metadata, such as sample temperature, chopper settings, slit positions *etc*. Amongst the benefits over traditional histogram measurements, the data may be re-reduced after the fact if, for example, a finer bin size is needed. Event mode also allows the data to be sliced or filtered after collection by time, temperature or any other variable recorded in the metadata (Granroth *et al.*, 2018 ▸).

An experiment is scripted using a table scan, where each line contains information about the desired parameters for a single run, such as temperature or wavelength, and the counting criteria. Each line is executed sequentially and additional table scans may be queued. POWGEN can count based on real time, which is useful for kinetics studies and other time-dependent effects. More commonly, POWGEN counts based on cumulative beam power, also known as proton charge or pcharge, in order to prevent temporary beam outages from affecting the data. More details on data collection are available on the POWGEN web site at https://neutrons.ornl.gov/powgen. For experiments where data are collected continuously during heating or cooling, a special ramping script is also available, which monitors the beam power and adjusts the temperature ramp rate as needed to ensure consistent statistics at all temperatures.

### 3.3. Instrument calibration and data reduction   

A successful powder diffraction measurement relies on being able to measure the peak positions and intensities accurately and being able to model them to extract structural parameters. In a TOF diffractometer, a white beam is chosen by the speed and frequency of the bandwidth choppers. Combining Bragg’s law and de Broglie’s equation gives us
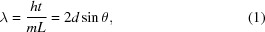
where λ is the wavelength, *m* is the mass of a neutron, *h* is Planck’s constant, *L* is the total flight path, θ is the scattering angle and *d* is the interatomic spacing in the crystal structure. Therefore,
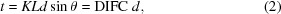
where DIFC = *KL*sinθ and *K* = 2*m*/*h* = 505.554 µs m^−1^ Å^−1^. Since the diffraction pattern collected in each individual pixel in a detector does not, in reasonable measurement time, detect enough neutrons for sufficient statistics for a Rietveld refinement, the process of time focusing is applied. This is done by applying corrections to the data on a per-pixel basis, before converting to *d* spacing and summing. Then the resulting spectrum is assigned an effective detector position, 2Θ, and secondary flight path, before being converted back to an effective TOF. This method of time focusing data is mathematically equivalent to the traditional method of multiplying the time of flight of the detector pixels with an offset value. Time focusing is necessary as Rietveld refinement programs (Rietveld, 1969 ▸) fit the data in TOF rather than *d* spacing. This results in the lining up of all the diffraction peaks in effective TOF. Previous studies have shown that failing to align the individual spectra well manifests as an unusual peak shape in the summed spectra (Olds *et al.*, 2018 ▸). Then the single summed spectrum is treated according to the equation scheme
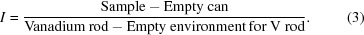
Here the empty measurements can also be smoothed to eliminate noise from the low counts of the empty measurements. If needed, users can also measure the empty cans for a longer time as the science dictates. The denominator in this equation accounts for the instrument response (such as incident spectrum, correction for solid angle and detector efficiency *etc*.) and is determined using a vanadium rod of diameter 6.35 mm, measuring for several hours to collect appropriate statistics. Before summing, a multiple scattering and absorption correction is applied to the vanadium measurement (Mildner & Carpenter, 1990 ▸). After time focusing, as described above, the Bragg peaks are stripped from the vanadium measurement, and a Butterworth low-pass filter for smoothing the data (Butterworth, 1930 ▸) is applied to decrease noise in the reduced data. Measurements for empty vanadium cans and the empty sample environment setting for the vanadium rod are also collected at the beginning of the cycle in the sample changer for the standard settings in Table 1 ▸. For all other sample environments, the empty-can measurements are carried out prior to sample measurements. The data are automatically reduced according to this scheme as individual raw data files (*i.e.* event files) are saved, but data can easily be combined or split after the measurement if the experiment requires it.

**Figure 5:**
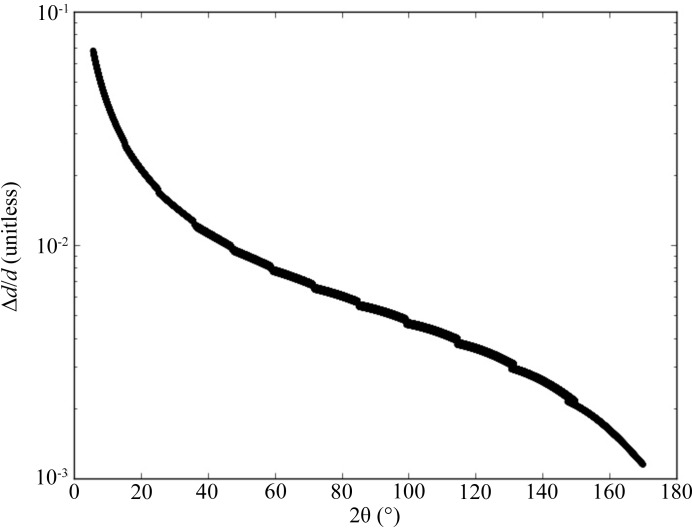
Instrument resolution as a function of the Bragg angle 2θ for a center wavelength of 0.8 Å using the high-resolution guide setting. The steps coincide with the various portions of the instrument and are from the ‘theoretical’ values calculated from the derivative of the Bragg equation. This demonstrates that the detector locations have a continuously varying resolution.

The upgraded instrument continues the theme of the initial design in having a matching peak resolution between the detectors as they sample different regions of reciprocal space. If the overlapping reflections from the pixels are of similar width, then summing them to generate an overall diffraction pattern will not have a negative effect on the resolution. The resolution function can be derived by taking the partial derivatives in equation (2) and can be written as
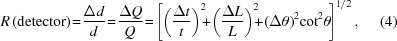
where *L* is the total flight path and θ is half the Bragg angle. This is plotted as a function of the Bragg angle in Fig. 5 ▸. In POWGEN, the locus of the detectors is chosen such that the overlapping reflections have comparable resolution. This is done as the *Q* coverage in each of the rectangular detector modules is relatively small owing to the short 1 Å bandwidth at 60 Hz. As a result, even though the cotθ term results in broader peaks in the forward scattering detectors, those reflections are not present in backscattering detectors where the peaks are sharper. This is the reason for the variable resolution as a function of *Q*. However, this mode of operation requires very high precision calibration of the TOF to *d*-space conversion for each individual pixel so that all of them can be seamlessly combined into one histogram. This is accomplished by measuring a diamond powder (40–60 µm, 99.9% metal basis) in the first frame where the center wavelength is 0.533 Å, so that a sufficiently large number of reflections is present in every single pixel. A total of 19 individual reflections are independently found in TOF and compared with the known *d* spacings of diamond powder. These are used to create a DIFC [as defined in equation (2)] for the individual pixels using the Gauss–Markov process. The *Mantid* software package is used for the calibration (Arnold *et al.*, 2014 ▸) of every single pixel (∼43 000 in the current instrument). The diffractometer constants are then stored in a file which is used for all data reduction. At present, second-order corrections are not carried out as we have not observed significant improvement from performing them; however, the algorithm exists to do so.

Several standard samples are measured at the beginning of each cycle for profile calibration. These are Si (SRM 660d), LaB_6_ (SRM 660b), Al_2_O_3_ (SRM 676a) and Na_2_Al_2_Ca_3_F_14_ (Courbion & Ferey, 1988 ▸). Several past publications have shown the formulation of peak-shape functions needed to model TOF data (Von Dreele *et al.*, 1982 ▸; David, 1986 ▸). Avdeev *et al.* (2007 ▸) have shown some of the additional terms needed for the empirical description of the peak position and profile. Owing to the somewhat different mode of operation at POWGEN as described earlier, we revisited these relationships and special routines were incorporated in *GSAS-II* (Toby & Von Dreele, 2013 ▸) for calibration of the instrument. The peak-shape parameters (Von Dreele *et al.*, 1982 ▸) α (rise coefficient), β (decay coefficient), σ (Gaussian contribution) and γ (Lorentzian contribution) define the shape of the rising and decaying exponentials and the Gaussian and Lorentzian components of the pseudo-Voigt function, respectively. We find that the *d* dependences of α, β and γ remain unchanged; however, an additional σ term improves the fits significantly. We also find that an additional term is needed to convert TOF, *T*, to *d* spacing. These relationships are given below:
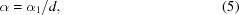


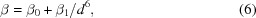


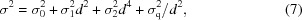


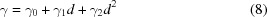
and
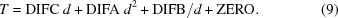
The calibration samples mentioned above are used to find these empirical parameters (α_1_, β_0_, β_1_, σ_0_, σ_1_, σ_2_, σ_q_, γ_0_, γ_1_, γ_2_, DIFC, DIFA, DIFB and ZERO) for the instrument for the commonly used chopper settings in Table 1 ▸ for both high-resolution and high-intensity guide settings. Some characteristic fits using these parameters are shown in Figs. 4 ▸(*c*)–4(*e*) for LaB_6_ (SRM 660b). The additional sample-dependent broadening needs to be refined separately. For the purposes of determining the resolution of the instrument, the sample-dependent broadening in LaB_6_ was fixed to the value determined from high-resolution synchrotron data collected at beamline 11BM at the Advanced Photon Source.

### 3.4. Intensity versus resolution   

The integrated intensity in a powder diffraction measurement at a TOF diffractometer can be used to estimate the required acquisition time. This Bragg intensity in a single time bin for a reflection τ is given by the formula
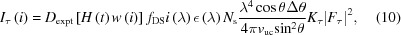
where *I*_τ_(*i*) is the number of neutron counts collected in the *i*th time bin for reflection τ(*n*), *D*_expt_ is the duration of the experiment (pulse), *H*(*t*) is the peak-shape function at relative time *t* = tof(*i*) − tof_τ_ (µs^−1^) [tof(*i*) being the TOF for the *i*th time bin], *w*(*i*) is the width of the *i*th time bin (µs), *f*_DS_ is the fraction of the Debye–Scherrer cone that intersects the pixel, *i*(λ) is the incident neutron flux at the sample (n cm^−2^ pulse^−1^ Å^−1^), ∊(λ) is the detector efficiency, *N*_s_ is the number of unit cells in the sample, λ is the wavelength (Å), 2θ is the Bragg angle for the pixel, 2*Δθ* is the 2θ coverage of the pixel (fraction of 1 rad), *v*_uc_ is the unit-cell volume (Å^3^), *F*_τ_ is the structure factor with the Debye–Waller factor included (cm) and *K*_τ_ = *E*_τ_(λ) [extinction] × *A*_τ_(λ) [attenuation] × *O*_τ_ [preferred orientation] × *m*_τ_ [reflection multiplicity].

It is immediately clear that there are very large gains in low-*Q* data due to the wavelength dependence of the diffracted intensity. If both high and low *Q* are needed and a time-dependent process is not being studied, users can easily use two different settings with different counting times (longer wavelength settings take less time to achieve the same level of statistics). Also, if we consider the resolution function stated in equation (4), by changing the wavelength one can relocate a certain Bragg peak to a different 2θ, which allows movement to a higher or lower resolution depending on the need of the science case. This mode of operation gives enormous flexibility in choosing the resolution and intensity.

**Figure 6:**
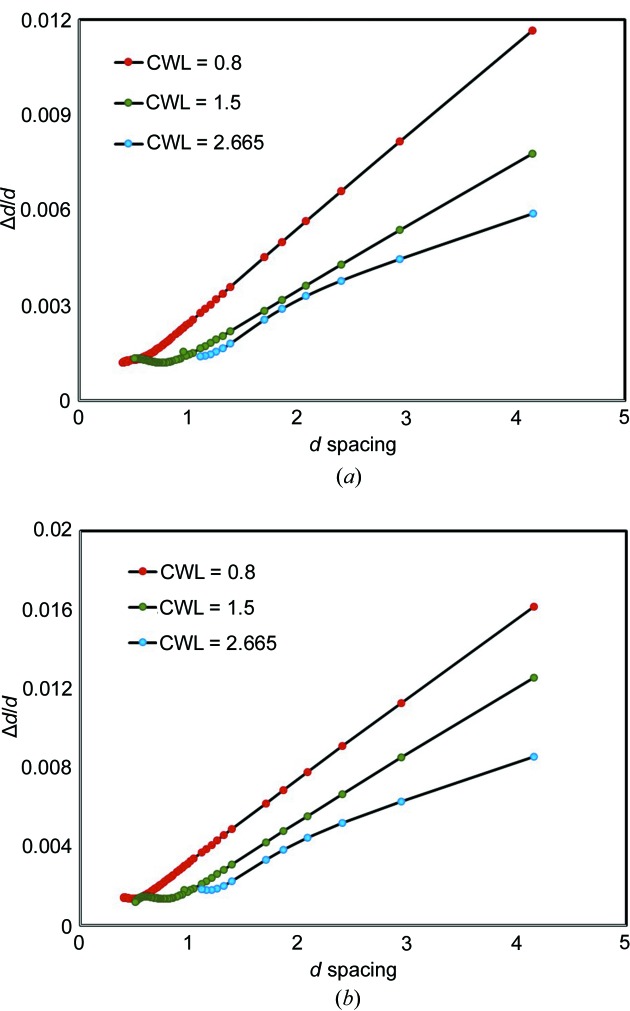
Resolution function Δ*d*/*d* (calculated from Δ*t*/*t*) using center wavelengths (CWL) of 0.8, 1.5 and 2.665 Å in the (*a*) high-resolution and (*b*) high-intensity mode. The lines are added as a guide to the eye. The data were collected from LaB_6_ (SRM 660b) with a frequency of 60 Hz and fitted using *GSAS-II*.

The resolution 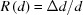 is plotted for three different settings in Fig. 6 ▸. In the backscattering detectors a resolution of 1 × 10^−3^ (obtained from FWHM calculations using *GSAS-II*) can be achieved, and even at low angles a very good resolution of 2 × 10^−3^ is maintained. Some of the resolution can be traded off for intensity by using the high-intensity guide setting. At center wavelengths of 0.8, 1.5 and 2.665 Å, one can expect intensity gains of 1.2, 1.8 and 2.2, respectively, from the high-resolution to high-intensity settings.

## 4. Sample environments   

Ancillary equipment is an integral part of a scattering instrument, especially at a powder diffractometer where most of the studies require control of temperature among other stimuli to follow the structure as a function of that external stimulus. Table 2 ▸ ▸ gives details of the standard equipment and relevant information required to plan an experiment at the instrument.

For electrochemical investigations, a potentiostat (±10 V, 400 or 240 mA with low-current channel) is available. Specialized cells for liquid or solid electrolyte cells are under development. Boron nitride (BN) sleeves mask parts of the cell outside of the desired sample. A custom sample stick may be used to place the cells inside the cryofurnace, which allows moderate heating (Song *et al.*, 2019 ▸).

Validating that the temperature of the sensor matches that of the sample is an important consideration, particularly for powder diffraction. Powder is a poor thermal conductor, and even sensors placed close to the sample may report a significantly different temperature than the powder itself. In order to verify the temperature of the cryostat and POWGEN auto changer (PAC), data are collected over the entire temperature range on a material with a well known thermal expansion curve, typically ZrW_2_O_8_ (Evans *et al.*, 1996 ▸). For high temperatures in the furnaces and cryofurnace, a mixture of MgO and Al_2_O_3_ is used. A parametric approach allows refinement of the temperature offset based on the lattice parameters (Reeber & Wang, 2000 ▸).

The upgrade allows access to a large *d*-spacing range (0.135–8.2 Å, for example) in a single frame. This is quite convenient as it eliminates the need for collecting time-consuming multiple frames. The wide 2Θ coverage also enables development of additional equipment to complement what is available at other diffractometers at the facility, such as a gas pressure cell, a 5 T magnet to make it feasible to study materials under a magnetic field and other equipment that is dictated by the needs of the user community.

## 5. Science examples enabled by upgrade   

POWGEN has an extremely versatile scientific portfolio at present. However, the upgrade has opened several additional avenues which are somewhat less explored. It is expected that, owing to the large *d* spacings which can be easily accessed using longer wavelength bands on POWGEN, it will be ideally suited for large-structure refinements such as metal organic frameworks (Trickett *et al.*, 2019 ▸) and zeolites (Latshaw *et al.*, 2016 ▸), and in the future, it is expected that one will be able to investigate gas absorption in these materials. The access to low *Q* also makes it an ideal instrument to study magnetic structures. Several such studies have already been done at the instrument (Li *et al.*, 2018 ▸; Hona *et al.*, 2018 ▸; Allred *et al.*, 2016 ▸), and their number is expected to continue to grow.

*In situ* measurements that follow the structure as a function of temperature (Murshed *et al.*, 2014 ▸), partial pressure of oxygen (Tamimi & McIntosh, 2014 ▸) and during materials synthesis (Abeysinghe *et al.*, 2018 ▸) are routine experiments at POWGEN. The upgrade simply allows faster data collection or use of a smaller sample. Recently there has been great interest in measuring samples that have a rather large absorption cross section due to exciting quantum phenomena, such as topological insulators, Weyl metals and Kitaev spin liquids which are observed in heavier transition-metal-containing compounds such as Ru, Rh, Os, Ir *etc.* (Sharma *et al.*, 2018 ▸). Owing to the high intensity offered by the source and the increased detector coverage from the upgrade, it is now feasible to study somewhat higher absorbing samples using thinner (3 mm diameter) or annular cans (1 mm annulus). However, data correction for such cases is still under development. The following are a few additional scientific examples that take advantage of the high intensity and resolution of this instrument.

### 5.1. Hydrogen-containing sample   

Hydrogen frequently plays a key role in molecular compounds and other compounds like minerals as a result of the natural inclusion of water. Owing to its low atomic number, it is very difficult to find hydrogen by traditional X-ray crystallography and thus neutrons have made a large contribution to locating hydrogen in crystal structures. Although single-crystal neutron diffraction has been the dominant technique for this purpose, with the high intensity and resolution afforded by modern powder diffractometers like POWGEN, one can also extract this information from neutron powder diffraction (NPD), particularly in cases where single crystals of sufficient size cannot be made.

Hydrogen has three isotopes. The most common are protium (^1^H) and deuterium (^2^H). The most abundant, protium (often called hydrogen), has a very large incoherent scattering cross section for neutrons and as a result contributes a large source of wavelength and energy-dependent background. On the other hand, deuterium has negligible incoherent scattering, making it ideal for diffraction work. The two isotopes protium and deuterium also have negative and positive scattering lengths of −3.742 and 6.674 fm, respectively. This means partial substitution in a site in the right ratio can cancel the nuclear density of a specific site. It is often expensive to deuterate materials and sometimes there are also changes in the chemistry depending on which isotope is used. As a result, being able to study the materials without deuteration is desirable. An extensive review has recently been published on the topic by Wilson *et al.* (2014 ▸).

**Figure 8:**
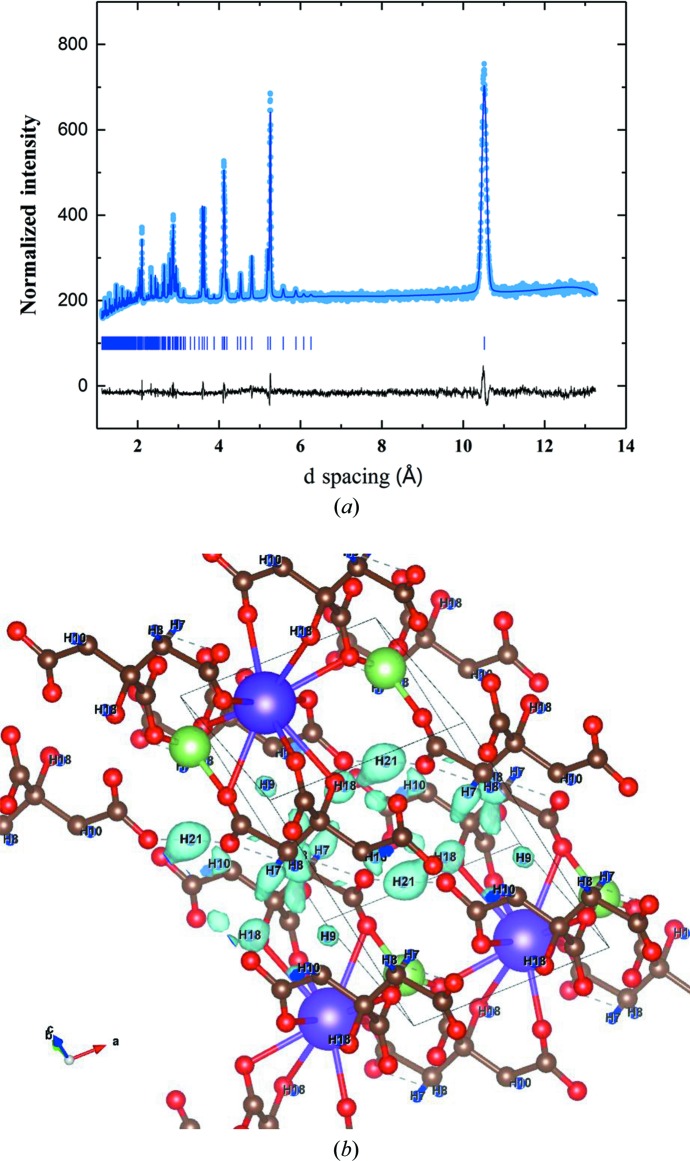
(*a*) Rietveld refinement of 1.22 g of LiKHC_6_H_5_O_7_ loaded in a 6 mm V can and measured for 2.6 h in the sample changer using a center wavelength of 2.665 Å at room temperature. Light-blue dots represent measured data, the royal-blue line is the model fit to the data and the black line is the difference curve. Tick marks show the locations of the expected Bragg peaks. (*b*) The Fourier density of the missing hydrogen is overlaid on the DFT-calculated hydrogen location.

Here we show one example of the quality of the data that can be collected with POWGEN on hydrogenous material for structure solution and refinement. Citrate salts have many applications in industry. Recently, a family of compounds with the general formula Li*M*HC_6_H_5_O_7_ (*M* = Li, Na, K and Rb) were prepared and their structures were solved by powder X-ray diffraction (XRD) measurements (Cigler & Kaduk, 2018 ▸). However, since XRD is not sensitive to hydrogen, their positions were calculated using density functional theory (DFT). A powder sample of LiKHC_6_H_5_O_7_ with a mass of 1.22 g was loaded in a 6 mm vanadium can and measured for 2.6 h in the sample changer using a center wavelength of 2.665 Å at room temperature. The DFT structure was used as a starting point and refining the structure gave a fit with a weighted *R* factor *R*_wp_ of 1.57. To test how sensitive the data are towards hydrogen, all the hydrogen atoms were removed from the refinement and a Fourier difference map was generated using *GSAS-II* (Toby & Von Dreele, 2013 ▸). All six hydrogen positions are clearly identifiable from these data, as seen in Fig. 8 ▸, which points to the feasibility of using NPD at POWGEN for locating hydrogen atoms in molecular compounds. Table 3 ▸ lists the coordinates of the atoms calculated via DFT along with those obtained from neutron refinement. Currently, samples with nearly 50 at.% hydrogen have been successfully measured on the instrument. Preliminary measurements have indicated that, in addition to locating the hydrogen atoms in molecular compounds, the structure of water in mineral samples can be resolved.

### 5.2. High-resolution garnet   

**Figure 9:**
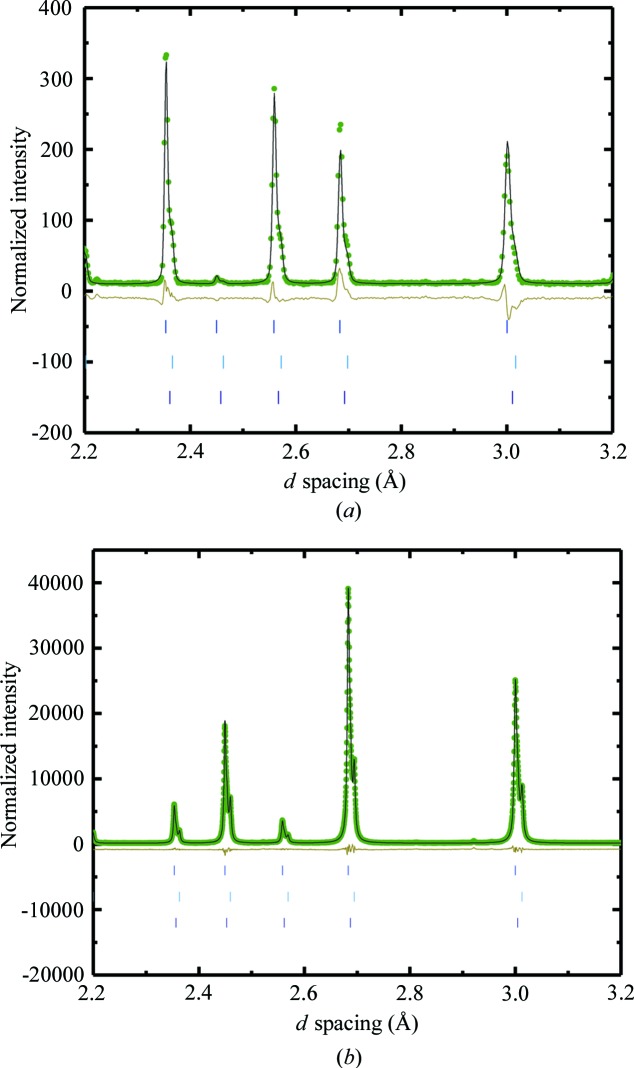
(*a*) Rietveld refinement of 1.09 g of andradite grossular Ca_3_(AlF)_2_Si_3_O_12_ from Crowsnest Pass loaded in a 6 mm V can and measured for 2.6 h in the sample changer using a center wavelength of 2.665 Å at room temperature. Light-green dots represent measured data, the black line is the model fit to the data and the olive line is the difference curve. Tick marks show the locations of the expected Bragg peaks. (*b*) Refinement of high-resolution data collected at beamline 11BM at APS from the same sample. The same *d*-spacing range is chosen for both plots to show a comparison of the resolution.

Garnets are a family of minerals widely used as gemstones. Garnet has the formula *A*_3_ *B*_2_(*X*O_4_)_3_ (*A* = Fe, Ca, Mg, Y, La or rare earth; *B* = Al, Fe, Ga, Ge, Mn, Ni or V; *X* = Si, Ge or Al), where *A*, *B* and *X* are cation sites with eight, six and four coordinated O ions, respectively. In nature the gemstones are generally silicates. This class of materials has recently attracted attention in the battery community as there are Li-stuffed garnet structures which show promise as a solid electrolyte material. A high conductivity of 10^−3^ S cm^−1^ has been observed in Li_6.4_La_3_Zr_1.4_Ta_0.6_O_12_ at 298 K. Depending on how the samples of this compound are synthesized and what the composition is, multiple cubic phases are frequently observed. However, the difference in the lattice parameters of these phases is so small that they can only be separated using the very high resolution diffraction available at synchrotron sources. A similar phenomenon has also been observed in natural garnets and is proposed to be the source of the observed birefringence in these compounds (Antao, 2013 ▸). In order to explore the resolution achievable on POWGEN, several samples of garnets were measured using two center wavelengths of 0.8 and 2.665 Å. X-ray data of these samples were available from the 11BM beamline at the Advanced Photon Source, which is one of the highest-resolution synchrotron powder diffractometers with a resolution of <1.4 × 10^−4^ Δ*d*/*d*. The highest resolution on POWGEN, like all other TOF diffractometers, is at the backscattering detectors. When the data are collected at 0.8 Å, the *d* spacings covered by backscattering are from 0.1 to 0.6 Å, where there is a high degree of overlap of reflections. However, when the data are collected at a longer wavelength, the longer-*d*-spacing reflections are now measured at the backscattering detectors and as a result the peak splitting from the multiple cubic phases is clearly observed in the pattern. Data collected in the same range at both beamline 11BM at APS and POWGEN at SNS are shown in Fig. 9 ▸. This example highlights that very small differences in lattice parameter can be detected at POWGEN, which is sometimes critical for following phase transitions and samples with mixtures of similar phases.

### 5.3. Total scattering   

**Figure 10:**
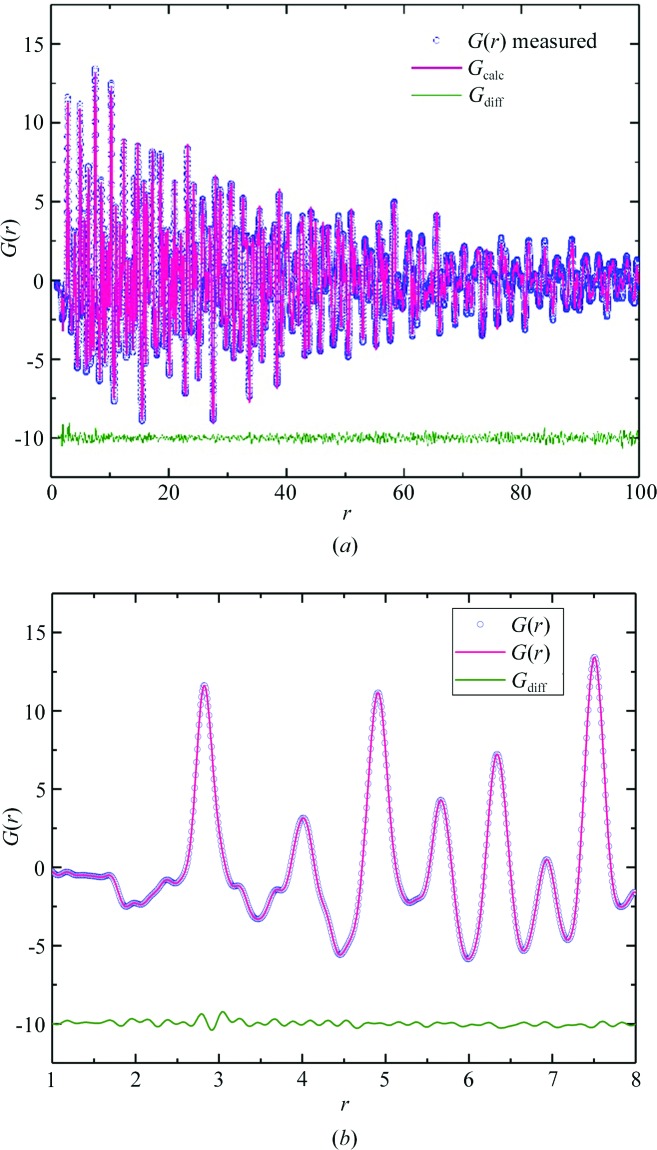
POWGEN PDF data measured using the high-intensity guide from a 6.4 g sample of BaTiO_3_ for a total measurement time of 2.5 h and generated with a *Q* range of 1–30 Å^−1^. *Q*_damp_ and *Q*_broad_ for 1–100 Å are 0.0118 and 0.0148 Å^−1^, respectively. Refinement results are shown for (*a*) a 1–100 Å fit using a tetragonal *P4mm* crystal structure as a model (*R*_wp_ = 6.68) and (*b*) a 1–8 Å fit using a rhombohedral *R*3*m* crystal structure as a model (*R*_wp_ = 4.12). Data are shown as circles, refinement results are shown as colored lines, and the difference curves are shown as solid green lines offset below the data and fits for clarity.

While total scattering is not the primary mission of this instrument, the shortest usable wavelengths provide quality data for PDF analysis. The extended *Q* range (in particular, the range of the 0.8 Å center wavelength setting) and increased measurement counts offered by POWGEN’s instrument upgrade have expanded opportunities for studies of disordered crystalline materials. Fig. 10 ▸ displays POWGEN PDF data collected for a 6.4 g sample of BaTiO_3_ for 2.5 h at room temperature with a 0.8 Å center wavelength and high-intensity guide setting. Data were transformed into real space using a *Q*_min_ of 1 Å^−1^ and a *Q*_max_ of 32 Å^−1^. This canonical ferroelectric material is well known at room temperature to possess local ordering that is rhombohedral in nature, while the long-range PDF and average structures are consistent with tetragonal symmetry (Senn *et al.*, 2016 ▸; Page *et al.*, 2010 ▸; Neilson & McQueen, 2015 ▸). The data have been fitted in the least-squares-based refinement program *PDFgui* (Farrow *et al.*, 2007 ▸) over 1–100 Å in real space. The quality of refinement matches or exceeds that available at other modern neutron total scattering beamlines. Factors influencing the quality of neutron TOF PDF data were recently reviewed (Olds *et al.*, 2018 ▸). POWGEN’s overall high resolution results in observable intensities exceeding 200 Å^−1^ in real space, while the influence of its near linear Δ*d*/*d* (equivalent to Δ*Q*/*Q*) resolution behavior displayed in Fig. 6 ▸, as a function of *d* spacing, is modeled straightforwardly with a Gaussian damping envelope available in the fitting software.

Local atomic structure studies and combined diffraction and PDF studies are primary missions of the Nanoscale Ordered Materials Diffractometer (NOMAD) (Neuefeind *et al.*, 2012 ▸) and the proposed DISCOVER beamlines at the SNS (Calder *et al.*, 2018 ▸). However, with its ability to measure PDF data up to over 100 Å in real space, POWGEN fills an important need for combined high-resolution Bragg scattering and disordered crystalline PDF studies, particularly when intermediate length scales (30–100 Å or more) are of interest. This has been demonstrated in our study of the nanoscale cation ordering in LiMn_1.5_Ni_0.5_O_4_ lithium-ion battery spinel samples, where the determined size of Mn and Ni cation ordered domains distinguishes samples with different charge/discharge rate capabilities (Liu *et al.*, 2016 ▸). In addition to cation or anion chemical short-range-order studies, potential applications include the study of charge and orbital ordered domains, detailed structural analysis of conversion or topochemical reactions, the nature of displacive phase transitions, and nanocrystallite studies including particle size/shape effects. Nonetheless, sample size requirements and the primary mission of the instrument provide some constraints on these potential applications. Expanded *Q* range (increased real-space resolution) and increased *Q* resolution (expanded real-space range) are possible at POWGEN by combining data from several center wavelength measurements. A more detailed characterization of POWGEN’s PDF capabilities will be presented in a future contribution.

## 6. Conclusions   

POWGEN is a third-generation design instrument that is an ideal compromise of resolution and intensity to study a wide variety of polycrystalline materials with extremely good resolution and yet a short counting time. The data collection time depends on the mass of the sample, the sample composition and crystallinity, the crystallographic symmetry, and the level of detail that is being sought from the data and can vary from minutes to hours. The versatility of the scientific applications can be appreciated by looking at the more than 400 publications that have resulted from the eight years of operation: https://neutrons.ornl.gov/powgen/publications. General user proposals are accepted twice a year and a rapid-access mail-in program also exists, where users can request 8 h of beamtime for standard experiments using the sample changer: https://neutrons.ornl.gov/powgen/mail-in.

## Figures and Tables

**Figure 7:**
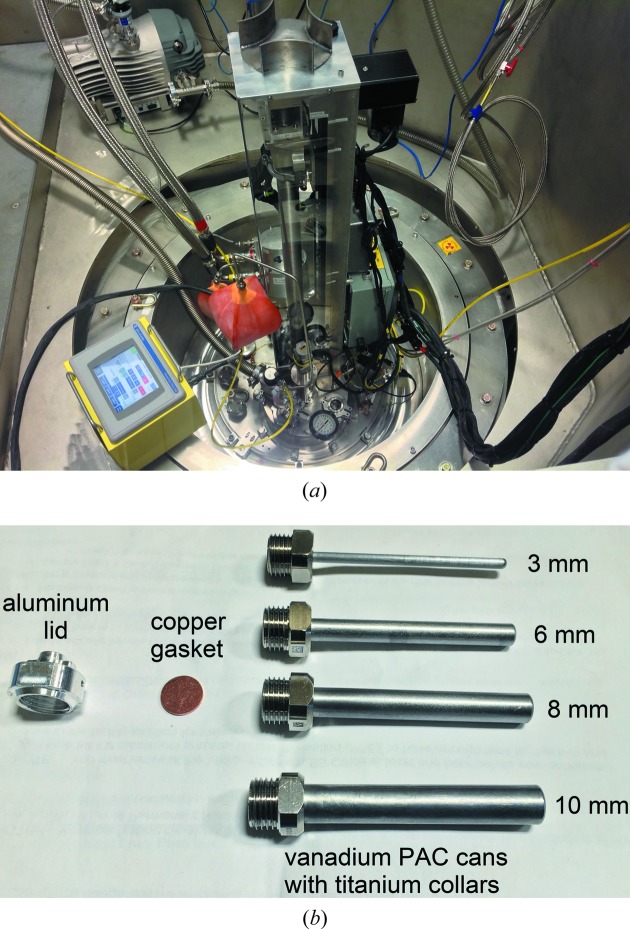
(*a*) POWGEN auto changer. This sample environment equipment was designed and built at SNS and enables loading of 24 samples in a carousel. These samples can then be changed, and the available temperature for measurement is 10–300 K. (*b*) Custom vanadium cans, with gasket and lid, designed for this changer. These cans are used for the mail-in program.

**Table 1:** Standard settings for data collection at POWGEN for which the instrument is calibrated

Frequency	λ_center_ (Å)	λ_min_ (Å)	λ_max_ (Å)	*d*_min_ (Å)	*d*_max_ (Å)	*Q*_min_ (Å^−1^)	*Q*_max_ (Å^−1^)
60	0.800	0.267	1.333	0.13	8.2	0.766	46.9
60	1.500	0.967	2.033	0.485	14.0	0.449	12.9
60	2.665	2.132	3.198	1.070	22.2	0.283	5.9
60	4.797	4.264	5.330	2.140	33.0	0.190	2.9
30	1.066	0.100	2.132	0.100	15.0	0.411	62.5
20	1.599	0.100	3.198	0.100	20.0	0.270	62.5
10	3.198	0.100	6.396	0.100	40.0	0.137	62.5

**Table 2:** The standard equipment and relevant information required to plan an experiment at the instrument

	Temperature range	Time to cool/heat	Sample holder	Other
POWGEN auto changer (PAC)	10–300 K.	45 min to cool from 300 to 10 K.	Specially designed V cans.	24 sample changer. The time for change is 8 min (Fig. 7 ▸).

Orange cryostat (Brochier, 1977 ▸)	2–300 K.	2 h to reach base temperature.	PAC cans.	Sample can be changed at 100 K.

Cryofurnace	5–500 K in cold stick. 30–750 K in hot stick.	2 h to cool from 750 to 300 K. 4 h to cool from 300 K to base temperature.	PAC cans for cold stick and V cans with Ti flange lid for hot stick.	Standard off-the-shelf closed-cycle refrigerator bought from Janis Research Company.

Vacuum and atmosphere furnaces (Niedziela *et al.*, 2017 ▸)	300–1473 K with V heating element. 300–1873 K with Nb heating element.	4–5 h to cool from 1473 to 473 K.	V can, BN lids and Mo screw.	Nb peaks are observed in forward and backscattering detectors when Nb heating element is used. As a result, the *Q* range is reduced.

Vacuum and atmosphere furnaces with gas insert (Kirkham *et al.*, 2018 ▸)	300–1123 K.	1.5 h to cool from 1123 to 473 K, with gas flow.	Quartz basket with one open end and frit on the other end.	Includes residual gas analyzer and pO_2_ sensor to measure the partial pressure of gas and detect incoming or outgoing gas. A wide variety of gasses are available. For specialized hazardous gas an additional review may be required.

**Table 3:** Fractional coordinates and isotropic displacement parameters (Å^2^) for Li*M*HC_6_H_5_O_7_ from XRD/DFT calculation and NPD on POWGEN The first row in each entry corresponds to the XRD/DFT calculation, the second corresponds to NPD on POWGEN, and the third row is the difference between the X-ray/DFT and NPD values. The unit-cell parameters and goodness of fit are also provided in the header of the table. Same-atom species are constrained to have the same thermal parameters (*U*_iso_). The position of Li is fixed for the refinement.Space group 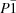. Lattice parameters: *a* = 6.2844 (6), *b* = 6.3148 (4), *c* = 10.8249 (17) Å (DFT), *a* = 6.2848 (4), *b* = 6.3142 (3), *c* = 10.8259 (10) Å (NPD), α = 93.059 (4), β = 102.951 (3), γ = 96.094 (2)° (DFT), α = 93.049 (3), β = 102.960 (2), γ = 96.093 (1)° (NPD). Goodness-of-fit parameters: *R*_wp_ = 2.56% (DFT); *R*_wp_ = 1.57 (NPD).

Atom	*x*	*y*	*z*	*U*_iso_ (Å^2^)
C1	1.1297	0.5120	0.3030	0.0099 (8)
	1.1334 (12)	0.4972 (14)	0.2932 (8)	0.0096 (7)
	0.0037	0.0148	0.0098	0.0003
C2	1.0220	0.6921	0.3505	0.0099 (8)
	1.0307 (13)	0.6930 (12)	0.3520 (8)	0.0096 (7)
	0.0087	0.0009	0.0015	0.0003
C3	0.8044	0.7464	0.2671	0.0099 (8)
	0.8230 (12)	0.7555 (13)	0.2666 (7)	0.0096 (7)
	0.0186	0.0091	0.0004	0.0003
C4	0.7260	0.9129	0.3512	0.0099 (8)
	0.7477 (12)	0.9280 (13)	0.3467 (8)	0.0096 (7)
	0.0217	0.0151	0.0046	0.0003
C5	0.5593	1.0569	0.2916	0.0099 (8)
	0.5612 (12)	1.0602 (12)	0.2852 (8)	0.0096 (7)
	0.0019	0.0032	0.0064	0.0003
C6	0.8475	0.8439	0.1456	0.0099 (8)
	0.8486 (12)	0.8225 (10)	0.1407 (6)	0.0096 (7)
	0.0011	0.0214	0.0049	0.0003
H7	1.1441	0.8338	0.3645	0.086 (4)
	1.1556 (32)	0.8050 (30)	0.3599 (17)	0.071 (4)
	0.0115	0.0288	0.0046	0.0150
H8	0.9965	0.6587	0.4444	0.086 (2)
	1.0319 (32)	0.6457 (32)	0.4529 (20)	0.071 (4)
	0.0354	0.0129	0.0084	0.0150
H9	0.8683	1.0210	0.4035	0.086 (4)
	0.8864 (28)	1.0670 (31)	0.3929 (16)	0.071 (4)
	0.0181	0.0459	0.0105	0.0150
H10	0.6582	0.8308	0.4232	0.086 (4)
	0.6962 (32)	0.8455 (30)	0.4299 (19)	0.071 (4)
	0.0380	0.0146	0.0066	0.0150
O11	1.2272	0.4066	0.3961	0.043 (2)
	1.2213 (17)	0.4122 (16)	0.3821 (10)	0.033 (1)
	0.0059	0.0056	0.0140	0.0100
O12	1.1355	0.4734	0.1906	0.043 (2)
	1.1159 (14)	0.4687 (16)	0.1853 (10)	0.033 (1)
	0.0196	0.0048	0.0053	0.0100
O13	0.5363	1.2099	0.3681	0.043 (2)
	0.5219 (15)	1.1799 (17)	0.3724 (9)	0.033 (1)
	0.0144	0.0300	0.0043	0.0100
O14	0.4561	1.0249	0.1774	0.043 (2)
	0.4896 (15)	1.0413 (15)	0.1760 (9)	0.033 (1)
	0.0335	0.0164	0.0013	0.0100
O15	0.9607	1.0242	0.1623	0.043 (2)
	0.9879 (14)	0.9871 (15)	0.9871 (15)	0.033 (1)
	0.0272	0.0371	0.0132	0.0100
O16	0.7692	0.7394	0.0397	0.043 (2)
	0.7653 (14)	0.7312 (17)	0.0361 (9)	0.033 (1)
	0.0039	0.0082	0.0036	0.0100
O17	0.6482	0.5615	0.2282	0.043 (2)
	0.6520 (15)	0.5738 (17)	0.2382 (10)	0.033 (1)
	0.0038	0.0123	0.0101	0.0100
H18	0.6337	0.4792	0.3003	0.086 (4)
	0.6306 (36)	0.5102 (35)	0.2968 (17)	0.071 (4)
	0.0031	0.0310	0.0035	0.0150
K19	0.2930	0.6951	−0.0061	0.081 (8)
	0.2920 (31)	0.7146 (30)	−0.0009 (16)	0.056 (6)
	0.0010	0.0195	0.0052	0.0052
Li20	0.7930	0.8114	0.8742	0.6
				0.27 (4)
H21	1.3404	0.3134	0.3710	0.086 (4)
	1.3641 (33)	0.2933 (34)	0.3777 (17)	0.071 (4)
	0.0238	0.0202	0.0067	0.0150

## References

[bb1] Abeysinghe, D., Huq, A., Yeon, J., Smith, M. D. & zur Loye, H. C. (2018). *Chem. Mater.* **30**, 1187–1197.

[bb2] Allred, J. M., Taddei, K. M., Bugaris, D. E., Krogstad, M. J., Lapidus, S., Chung, D. Y., Claus, H., Kanatzidis, M. G., Brown, D. E., Kang, J. Y., Fernandes, R. M., Eremin, I., Rosenkranz, S., Chmaissem, O. & Osborn, R. (2016). *Nat. Phys.* **12**, 493–498.

[bb3] Antao, S. M. (2013). *Powder Diffr.* **28**, 281–288.

[bb4] Arnold, O., Bilheux, J. C., Borreguero, J. M., Buts, A., Campbell, S. I., Chapon, L., Doucet, M., Draper, N., Ferraz Leal, R., Gigg, M. A., Lynch, V. E., Markvardsen, A., Mikkelson, D. J., Mikkelson, R. L., Miller, R., Palmen, K., Parker, P., Passos, G., Perring, P. F., Peterson, T. G., Ren, S., Reuter, M. A., Savici, A. T., Taylor, J. W., Taylor, R. J., Tolchenov, R., Zhou, W. & Zikovsky, J. (2014). *Nucl. Instrum. Methods Phys. Res. A*, **764**, 156–166.

[bb5] Avdeev, M., Jorgensen, J., Short, S. & Von Dreele, R. B. (2007). *J. Appl. Cryst.* **40**, 710–715.

[bb6] Brochier, D. (1977). ILL Technical Report No. 77/74. Institut Laue–Langevin, Grenoble, France.

[bb7] Butterworth, S. (1930). *Exp. Wireless Wireless Eng.* **7**, 536–541.

[bb8] Calder, S., An, K., Boehler, R., Dela Cruz, C., Frontzek, M., Guthrie, M., Haberl, B., Huq, A., Kimber, S. A., Liu, J., Molaison, J. J., Neuefeind, J., Page, K., Dos Santos, A. M., Taddei, K. M., Tulk, C. A. & Tucker, M. G. (2018). *Rev. Sci. Instrum.* **89**, 092701. 10.1063/1.5033906 30278771

[bb9] Cigler, A. J. & Kaduk, J. A. (2018). *Acta Cryst.* C**74**, 1160–1170. 10.1107/S2053229618012597 30284983

[bb10] Courbion, G. & Ferey, G. (1988). *J. Solid State Chem.* **76**, 426–431.

[bb11] Dalesio, L. R., Kraimer, M. R. & Kozubal, A. J. (1991). *Proceedings of the 2nd International Conference on Accelerator and Large Experimental Physics Control Systems (ICALEPCS1991)*, 11–15 November 1991, Tsukuba, Japan, p. 278. Tsukuba: KEK.

[bb12] David, W. I. F. (1986). *J. Appl. Cryst.* **19**, 63–64.

[bb13] Evans, J. S. O., Mary, T. A., Vogt, T., Subramanian, M. A. & Sleight, A. W. (1996). *Chem. Mater.* **8**, 2809–2823.

[bb14] Farrow, C. L., Juhás, P., Liu, J. W., Bryndin, D., Božin, E. S., Bloch, J., Proffen, T. & Billinge, S. J. L. (2007). *J. Phys. Condens. Mater.* **19**, 335219. 10.1088/0953-8984/19/33/335219 21694142

[bb15] Granroth, G. E., An, K., Smith, H. L., Whitfield, P., Neuefeind, J. C., Lee, J., Zhou, W., Sedov, V. N., Peterson, P. F., Parizzi, A., Skorpenske, H., Hartman, S. M., Huq, A. & Abernathy, D. L. (2018). *J. Appl. Cryst.* **51**, 616–629.

[bb16] Hona, R. K., Huq, A. & Ramezanipour, F. (2018). *Mater. Res. Bull.* **106**, 131–136.

[bb17] Huq, A., Hodges, J. P., Gourdon, & O., Heroux, L. (2011). *Z. Kristallogr. Proc.* **1**, 127–135.

[bb50] Huq, A. & Rennich, G. (2016). SNS Report No. 107100000-DC0001-R02. Spallation Neutron Source, Oak Ridge National Laboratory, TN, USA.

[bb19] Kasemir, K. (2007). *Proceedings of the 11th International Conference on Accelerator and Large Experimental Physics Control Systems (ICALEPCS2007)*, 15–19 October 2007, Knoxville, Tennessee, USA, p. 692. Geneva: JACoW.

[bb20] Kirkham, M. J., Heroux, L., Ruiz-Rodriguez, M. & Huq, A. (2018). *Rev. Sci. Instrum.* **89**, 092904. 10.1063/1.5031432 30278698

[bb21] Latshaw, A. M., Chance, W. M., Morrison, G., zur Loye, K. D., Wilkins, B. O., Smith, M. D., Whitfield, P., Kirkham, M. J., Stoian, S. A. & zur Loye, H. C. (2016). *Angew. Chem. Int. Ed.* **55**, 13195–13199. 10.1002/anie.201607800 27652577

[bb22] Li, M. R., Stephens, P. W., Croft, M., Deng, Z., Li, W., Jin, C., Retuerto, M., Hodges, J. P., Frank, C. E., Wu, M., Walker, D. & Greenblatt, M. (2018). *Chem. Mater.* **30**, 4508–4514.

[bb23] Liu, J., Huq, A., Moorhead-Rosenberg, Z., Manthiram, A. & Page, K. (2016). *Chem. Mater.* **28**, 6817–6821.

[bb24] Mildner, D. F. R. & Carpenter, J. M. (1990). *J. Appl. Cryst.* **23**, 378–386.

[bb25] Murshed, M. M., Mendive, C. B., Curti, M., Nénert, G., Kalita, P. E., Lipinska, K., Cornelius, A., Huq, A. & Gesing, T. M. (2014). *Mater. Res. Bull.* **59**, 170–178.

[bb26] Neilson, J. R. & McQueen, T. M. (2015). *J. Appl. Cryst.* **48**, 1560–1572. 10.1107/S1600576715016404 PMC4603272 26500465

[bb27] Neuefeind, J., Feygenson, M., Carruth, J., Hoffmann, R. & Chipley, K. (2012). *Nucl. Instrum. Methods Phys. Res. B*, **287**, 68–75.

[bb28] Niedziela, J. L., Mills, R., Loguillo, M. J., Skorpenske, H. D., Armitage, D., Smith, H. L., Lin, J. Y. Y., Lucas, M. S., Stone, M. B. & Abernathy, D. L. (2017). *Rev. Sci. Instrum.* **88**, 105116. 10.1063/1.5007089 29092522

[bb29] Olds, D., Saunders, C. N., Peters, M., Proffen, T., Neuefeind, J. & Page, K. (2018). *Acta Cryst.* A**74**, 293–307. 10.1107/S2053273318003224 29978841

[bb30] Page, K., Proffen, T., Niederberger, M. & Seshadri, R. (2010). *Chem. Mater.* **22**, 4386–4391.

[bb31] Peterson, P. F., Campbell, S. I., Reuter, M. A., Taylor, R. J. & Zikovsky, J. (2015). *Nucl. Instrum. Methods Phys. Res. A*, **803**, 24–28.

[bb32] Radaelli, P. G. (1998). *Proceedings of the 14th Meeting of the International Collaboration on Advanced Neutron Sources*, Vol. II, pp. 159–168. Argonne National Laboratory.

[bb33] Reeber, R. R. & Wang, K. (2000). *MRS Proc.* **622**, T6.35.1.

[bb34] Rietveld, H. M. (1969). *J. Appl. Cryst.* **2**, 65–71.

[bb35] Senn, M. S., Keen, D. A., Lucas, T. C. A., Hriljac, J. A. & Goodwin, A. L. (2016). *Phys. Rev. Lett.* **116**, 207602. 10.1103/PhysRevLett.116.207602 27258883

[bb36] Sharma, N., Mbarki, M., Zhang, Y., Huq, A. & Fokwa, B. T. (2018). *Angew. Chem. Int. Ed.* **57**, 10323–10327. 10.1002/anie.201804841 29892987

[bb37] Song, B., Veith, G. M., Park, J., Yoon, M., Whitfield, P., Kirkham, M. J., Liu, J. & Huq, A. (2019). *Chem. Mater.* **31**, 124–134.

[bb38] Tamimi, M. A. & McIntosh, S. (2014). *J. Mater. Chem. A*, **2**, 6015–6026.

[bb39] Toby, B. H. & Von Dreele, R. B. (2013). *J. Appl. Cryst.* **46**, 544–549.

[bb40] Trickett, C. A., Osborn Popp, T. M., Su, J., Yan, C., Weisberg, J., Huq, A., Urban, P., Jiang, J., Kalmutzki, M. J., Liu, Q., Baek, J., Head-Gordon, M. P., Somorjai, G. A., Reimer, J. A. & Yaghi, O. M. (2019). *Nat. Chem.* **11**, 170–176. 10.1038/s41557-018-0171-z 30455431

[bb41] Von Dreele, R. B., Jorgensen, J. D. & Windsor, C. G. (1982). *J. Appl. Cryst.* **15**, 581–589.

[bb42] Wang, C. L. (2016). *Rev. Sci. Instrum.* **87**, 053303. 10.1063/1.4949496 27250410

[bb43] Wang, C. L., Crow, L., Funk, L. L., Hannan, B. W., Hodges, J. P. & Riedel, R. A. (2015). IEEE Nuclear Science Symposium and Medical Imaging Conference (NSS/MIC).

[bb44] Wilson, C. C., Henry, P. F., Schmidtmann, M., Ting, V. P., Williams, E. & Weller, M. T. (2014). *Crystallogr. Rev.* **20**, 162–206.

